# A Few Suggestions as to Care and Treatment of the Horse’s Foot1Read before the Veterinary Medical Association of New Jersey, September 13, 1900.

**Published:** 1901-05

**Authors:** E. L. Loblein

**Affiliations:** New Brunswick, N. J.


### A FEW SUGGESTIONS AS TO CARE AND TREATMENT OF THE HORSE'S FOOT.[1]

By E. L. Loblein, V.S.,
NEW BRUNSWICK, N. J.

My observations with the colt during the first year of its life have forced the conclusion on me that care and treatment during that time have much to do with the conformation of the foot when the colt has matured, and faulty conformation of the foot is responsible for so many causes of lameness, both in the foot, in the limb above it, and also in opposite limbs, that it at once becomes apparent to the ordinary observer that an important factor in locomotion is a well-conformed foot.

The colt's foot when left to itself shows peculiar tendencies to grow in different directions according to the way the weight of the body is brought to bear on the part of the foot covering the ground surface. As an example : If two colts are turned loose in a pasture field, and six months later, if the feet have received no attention, it will be found that one colt's feet may be very long at the toe, giving the horn a slant and curve at the heel, which, in after life, will surely when shod produce sore or tender heels, and, finally, disease of the alæ or retrossal processes of the os pedis—a condition that more frequently occurs than is commonly believed, far more frequently than the old and almost worn-out diagnosis of navicular disease, which used to be the invariable diagnosis by exclusion ; whenever the veterinarian failed to discover any other cause for lameness it used to be navicular disease, but it pleases me to note that the fashion is dying out. Numerous other conditions arise from this malformation by the long heel and long toe. Very often the ankles are compelled to bear too much strain, as will be observed when viewed from either a physical or mechanical stand-point, as exists in the condition cited, and we all know how frequently ring-bones arise in colts from the same existing condition in the foot, and all can be obviated by the use of an ordinary rasp in the hands of an intelligent caretaker by keeping the toes short and distributing the weight evenly over the foot, which in after life with proper shoeing the foot is likely to grow in the directions and at the angles it has been induced to grow in early life.

In the other colt the opposite condition may be seen, and that

[1] Read before the Veterinary Medical Association of New Jersey, September 13, 1900.

is, for the toe to become worn and broken off and the heel to grow disproportionately high and steep, which also has its bad effects on the foot in after life. If the horn grows too perpendicular there becomes an uneven pressure on the os pedis and surrounding tissues, and the natural angles of the phalanges are destroyed, and consequent imperfect articulation ensues. This is a frequent source of lameness, as evidenced by periostites of bones adjacent to articulations. All this can be obviated by maintaining the proper angles by the use of rasp on ground surface of foot producing the tendency to grow a normal shaped foot.

When the time comes for our colts to be shod more care than ever should be exercised. My views are in no way radical or original on this point, as they are largely an indorsement of the views set forth by others who long claim to be authority on this subject. One point I want to emphasize as important and that is, that the shoes be light and never thick at the heels; at least the dressing of the foot and fittings of the shoe should be such as to allow the frog to come in contact with the ground all the time, as the colt's foot is sure to suffer as soon as the frog is removed from the ground.

One of the most important faculties for us to cultivate is true sight, and by constant application with the use of calipers and rule we will in time attain that proficiency in this one thing that will be of great use and assist us in diagnosticating lameness. Most of us could recall amusing incidents of our errors in discovering the source of lameness. Take, for instance, many road horses; just the slightest bruise on the metacarpal bones will produce periostitis and consequent lameness, and all traceable to an uneven foot, destroying the natural perpendicular or straight line of the limb during locomotion, bringing the leg in the way of the foot, not the foot in the way of the leg, as when one foot is perfectly level and the other uneven it is the leg resting on the uneven foot that is injured, showing that it got in the way of the foot being moved in its natural straight way. Ask for proof. Shoe the foot level the offence stops, and lameness—if injury is not too severe—soon passes away.

We have rambled on beyond the shoeing of a colt's feet, and come to the time when he goes to work, gets fed strong and driven hard, and there is every tendency to produce congestion of blood-vessels of the foot from so many causes. Our great object is to obviate or minimize the several factors that predispose the foot to become congested, and here, again, I say maintain your frog pressure, and when that bears its due share of weight and concussion brought to bear while in motion, either rapid or slow, the other parts will not be compelled to bear an undue portion of weight and concussion. Here, when the frog is not sufficiently developed, the well-adjusted bar-shoe is of inestimable benefit. If the running horse could wear bars without slipping, as they certainly do, it would prevent what is a great source of trouble with this particular class of horses, and that is spreading of the walls due to the bursting of the sole at the junction of the wall and sole. I have known several to do all their preparatory work in bar shoes, but when the bars were removed and they were run in plates the weak foot would frequently burst open. This condition is so seldom seen in any other class of horses that only those who have had experience with the runner have probably met with it.

The conditions conducive to health or disease of the foot leads us to another part of the anatomy—that is, the stomach—which at first sight may appear a digression from the subject, but such is really not the case. I assert with confidence that the diet—and the consequent maintenance of a healthy condition of the stomach—has much to do with the health of the foot. If an acute inflammation of the stomach—so frequently is the cause of acute laminitis of a metastatic nature—probably evident through the history of the case, deductions drawn from symptoms, and often direct evidence, having treated the animal the night before for gastritis; why, then, will not what we often meet with—a mild attack of inflammation of the stomach—produce the same condition in the feet not noticeable at the time, but plainly so when the same condition has existed for some time. In any other part of the animal a similar form of inflammation would soon pass away, but the anatomical formation and peculiar distribution of bloodvessels preclude the likelihood of inflammation at this point passing away without leaving some bad effects. The conditions cited set forth the importance of proper diet, and are proofs that the horse cannot transgress this law as often and with the impunity of his master. I think the majority of us would be hopeless cripples if abuse of the stomach had the same effect to the same extent on us.

I dare say that most of us have noticed that when the grain was taken from a horse and he was turned to pasture the feet lose brittleness and grow tough and strong. This is not due entirely to the moisture obtained from the wet grass and damp ground, but is partly due to the healthy condition of the stomach and absence of inflammation in the feet.

To go into all the causes would take too long, for the subject is an inexhaustible one, but heredity must not be overlooked. Heredity influences the general conformation, strength, size, and vascularity. The old adage that "like begets like" is very true in this case. So it is not surprising to see the offspring of a sire or dam who has bad feet showing a predisposition to the same condition. Nevertheless, I think it wrong to look on hereditary influences as inevitable. Take the young offspring and employ every means to obviate and overcome the tendency to follow in its parents' footsteps. I think the expression applicable here. The result will be a strong and comparatively healthy foot, and the offspring of that animal will probably be much improved in comparison to its foreparents, showing that heredity even in this can be successfully combatted if proper means are employed.

Of the artificial appliances we all know many to be beneficial, but there is one of the latest that I deprecate the necessity for using, and that is pads on soles and frog. I have failed to find where they have been used any length of time that they have not produced a dry rot of sole and frog, and in most cases the frog never regains its previous healthy state. Asphalt pavements seem to render this inevitable in some cities. Aside from this it is doubtful if they are ever of any permanent benefit. The use of hoof ointment, I think, is a dirty humbug. Water properly applied is of more permanent benefit than any other application that I have been lucky enough to meet with.

